# Moderating Role of Cigarette Smoking on the Efficacy of tDCS in the Treatment of Negative and Cognitive Symptoms of Schizophrenia: Results from a Randomized Clinical Trial

**DOI:** 10.3390/brainsci16020186

**Published:** 2026-02-03

**Authors:** Jacopo Lisoni, Gabriele Nibbio, Mattia Ardesi, Antonio Baglioni, Lorenzo Bertoni, Francesco Bezzi, Camilla Agnese Carolina Cicolari, Federica Frigerio, Michela Gregorelli, Paola Miotto, Giacomo Deste, Stefano Barlati, Antonio Vita

**Affiliations:** 1 Department of Mental Health and Addiction Services, ASST Spedali Civili of Brescia, Piazzale Spedali Civili 1, 25123 Brescia, Italy; miottopaola@yahoo.it (P.M.); stefano.barlati@unibs.it (S.B.); antonio.vita@unibs.it (A.V.); 2 Department of Clinical and Experimental Sciences, University of Brescia, Viale Europa 11, 25123 Brescia, Italy; gabriele.nibbio@gmail.com (G.N.); m.ardesi007@unibs.it (M.A.); a.baglioni@unibs.it (A.B.); l.bertoni005@unibs.it (L.B.); f.bezzi002@unibs.it (F.B.); cicolaricamilla@gmail.com (C.A.C.C.); f.frigerio003@studenti.unibs.it (F.F.); gregorelli@live.it (M.G.); giacomodeste@me.com (G.D.); 3 Department of Mental Health and Addiction Services, ASST Vallecamonica, Via Nissolina 2, 25040 Brescia, Italy

**Keywords:** tDCS, schizophrenia, negative symptoms, cognitive symptoms, cigarette smoking, neuroplasticity, oxidative stress

## Abstract

**Background:** Transcranial Direct Current Stimulation (tDCS) has shown potential in improving negative symptoms (NS) and Cognitive Impairment Associated with Schizophrenia (CIAS). However, heterogeneity in stimulation protocols and sample characteristics limit definitive conclusions regarding tDCS effectiveness in schizophrenia. Given the detrimental effects of cigarette smoking, particularly on cognition, this study explored the role of cigarette smoking as a modifiable individual factor potentially contributing to methodological heterogeneity by evaluating tDCS effects on NS and CIAS in Smoker (SM) and Non-Smoker (NoSM) patients. **Methods**: Post hoc analyses of a double-blind RCT were performed on 50 patients, randomized to 2 mA active or sham-tDCS (15 weekday sessions) with bilateral bipolar-nonbalanced prefrontal placement. The sample was divided according to the smoking status, consisting of 28 SM and 22 NoSM. Separate one-way analyses of covariance (ANCOVA) were performed within each subgroup to assess changes over time between treatment conditions. Clinical outcomes included Positive and Negative Symptoms Scale (PANSS), Brief Assessment of Cognition in Schizophrenia (BACS), Clinical Global Impression (CGI) and Calgary Depression Scale for Schizophrenia (CDSS) total scores. **Results**: SM exhibited baseline lower cognitive scores in verbal memory, motor speed and working memory domains. NS improved in both SM and NoSM with large effect size. Significant improvement in CIAS, specifically in working memory and verbal fluency, were found exclusively in NoSM. **Conclusions**: Cigarette smoking appeared to limit tDCS effectiveness in improving CIAS but not NS in schizophrenia. We suggested that the *neurotoxic milieu* linked to chronic exposure to neurotoxins of cigarette smoking could be responsible for these effects, counterbalancing the neuroprotective effects of tDCS. Further studies are warranted to replicate these findings.

## 1. Introduction

Schizophrenia is a severe and debilitating psychiatric condition that significantly impairs patients’ quality of life and well-being. Indeed, schizophrenia is characterized by poor psychosocial functioning, high levels of internalized stigma and substantial economic costs for mental health providers [1,2,3]. Among people living with schizophrenia (PLWS), cognitive impairments, closely followed by negative symptoms (NS), are consistently recognized as the main determinants of poor psychosocial and functional impairment [4,5,6]. Considering their clinical relevance and impact, the cognitive impairments observed in PLWS are internationally labeled under the umbrella definition of Cognitive Impairment Associated with Schizophrenia (CIAS) [7,8], and include impairments in processing speed, attention, working memory, verbal and visual learning and memory, verbal fluency, executive functions, and social cognition [9,10,11]. NS are currently conceptualized as two distinct dimensions: avolition/apathy, including the domains avolition, asociality and anhedonia; and diminished expression, including the domains blunted affect and alogia [12,13].

Despite significant advances in understanding the impact of CIAS and NS on clinical and functional outcomes, these symptom dimensions continue to represent major unmet therapeutic needs in the management of schizophrenia, constituting key barriers to functional recovery [14,15]. Notably, no pharmacological agents have been specifically approved for the treatment of CIAS [16]. Similarly, although the European Psychiatric Association guidelines recommend the use of second-generation antipsychotics (SGAs) due to their favorable cognitive profile compared to first-generation antipsychotics (FGAs) [11], no clear evidence supports the superiority of any single SGA over others in improving NS [13,17].

While no single model on the pathogenesis of CIAS and NS in PLWS can currently be considered definitive, recent evidence indicates that alterations in the balance between excitatory and inhibitory (E/I) neurons within cortical microcircuits represent a core pathophysiological feature [18,19]. This balance results from a complex interaction between different neural populations and involves different neurotransmitter pathways, including the dopaminergic, glutamatergic, γ-Aminobutyric acid (GABA)-ergic and cholinergic pathways [19,20,21,22]. It has been hypothesized that loss or aberrant functioning of parvalbumin-positive GABAergic interneurons may play a key role; however, it remains unclear whether this alteration reflects a primary pathogenic mechanism or a secondary consequence of other underlying abnormalities [23,24].

The role of the nicotinic system in the pathophysiology of schizophrenia and of CIAS has widely been recognized [25,26,27]. Cigarette smoking is highly prevalent among PLWS, with rates estimated at approximately 65% [28]. Indeed, acute nicotine administration has been shown to improve sensory gating and cognitive performance in schizophrenia [29]; accordingly, PLWS often report anxiety reduction and improved concentration as primary motivations for tobacco use [30,31]. These observations have led to the hypothesis that smoking in schizophrenia may represent a form of self-medication [30,32,33,34]. However, despite the transient cognitive enhancement associated with acute nicotine exposure, chronic tobacco smoking does not provide long-term cognitive improvement in PLWS [35]. Moreover, substantial evidence showed that cigarette smoking not only contributes to premature mortality from non-communicable diseases in PLWS [36] but may also exacerbate CIAS [37,38,39].

While currently available pharmacological treatments appear to exert only very limited effects on CIAS and NS, several non-pharmacological interventions have demonstrated clinically meaningful benefits on these outcomes [11,13,40]. Among these, Non-Invasive Brain Stimulation (NIBS) techniques—including repetitive transcranial Magnetic Stimulation, rTMS, and transcranial Direct Current Stimulation (tDCS)—are based on the modulation of brain activity through magnetic or electric induction and may represent promising strategies to improve these hard-to-treat symptom domains [40,41,42,43,44,45,46,47]. Specifically, tDCS is a well-tolerated and low-cost intervention that delivers low-amplitude direct currents through electrodes applied to the scalp. tDCS modulates cortical excitability in a non-focal way through polarity-dependent shifts of neuronal membrane potentials [47]. Moreover, tDCS has been associated with neuroplastic effects linked to the calcium-dependent modulation of synaptic plasticity in N-methyl-D-aspartate (NMDA) glutamatergic neurons and the regulation of GABAergic activity through mechanisms similar to long-term potentiation (LTP) and long-term depression (LTD) [48,49]. Furthermore, the clinical effects of tDCS have also been linked to its capacity to enhance glutamatergic signaling while attenuating cortical GABAergic transmission, as well as to modulate monoaminergic release [48,49,50]. Additionally, growing evidence from preclinical and neurological studies suggests that tDCS exerts multiple neuroprotective effects (including anti-inflammatory, anti-apoptotic, anti-oxidative, and pro-angiogenic actions) through the modulation of microglial activation and inflammatory pathways, enhancement of pro-survival signaling, reduction in oxidative stress, and promotion of vascular remodeling [49,50,51,52,53].

As for schizophrenia, recent meta-analytic evidence has shown that tDCS provides significant benefits in the treatment of NS and also appears promising for improving CIAS, particularly in the working memory domain [41,42,54,55,56]. Notably, prefrontal tDCS with the anodic stimulation of the left Dorsolateral Prefrontal Cortex (DLPFC) represents the configuration associated with the most robust effects on both NS and CIAS [42,43,57,58,59].

The main limitation regarding the efficacy of tDCS in schizophrenia relates to the substantial heterogeneity of stimulation protocols. By contrast, little attention has been devoted to the examination of patients’ clinical and personal characteristics. From this perspective, one approach to address such heterogeneity may be to focus on modifiable individual factors whose impact on clinical outcomes is well-established. Among these, cigarette smoking is particularly relevant, given its well-documented long-term detrimental effects, especially on cognitive functioning [60,61]. However, available evidence indicates that cigarette smoking status has rarely been considered in tDCS studies and has been infrequently examined as a potential moderator of treatment effects in meta-analytic assessments. Indeed, while its effects on NS and CIAS remain unclear, only preliminary evidence suggests that cigarette smoking may attenuate the effects of tDCS on positive symptoms [62]. Brunelin and colleagues reported that smoking status negatively impacted the efficacy of 10 sessions of 2 mA fronto-temporal tDCS on treatment-resistant auditory verbal hallucinations (AVH) in a small sample of PLWS (*n* = 16): while non-smoker patients showed a robust reduction in AVH severity, smoker patients exhibited no significant clinical improvement, with higher response rates in non-smokers if compared to smokers [62]. On the other hand, evidence on tDCS and rTMS effects on cigarette consumption and craving in PLWS remains largely inconclusive and inconsistent, as highlighted in a recent systematic review [63].

To advance our understanding of factors that may mitigate tDCS efficacy, this study aimed to investigate whether cigarette smoking status could moderate the effects of a bilateral bipolar non-balanced prefrontal tDCS protocol on NS and CIAS, through dedicated secondary analyses of a randomized controlled trial (RCT) [64,65]. Given its detrimental effects on CIAS, the primary research hypothesis was that cigarette smoking might limit the effects of tDCS, particularly in the treatment of CIAS. In addition, we aimed to determine whether Smoker patients (SM) exhibited more pronounced CIAS compared to Non-smokers (NoSM), thereby indirectly supporting the smoking self-medication hypothesis.

## 2. Materials and Methods

### 2.1. Study Design, tDCS Protocol and Assessment

For the present study, we conducted post hoc secondary analyses derived from a randomized, double-blind, sham-controlled study (active-tDCS versus sham-tDCS) that was previously published [64]. The original RCT was designed to evaluate the effects of a multi-session prefrontal tDCS protocol to primarily improve NS and CIAS in a sample of PLWS. A detailed description of the original RCT is provided in Lisoni et al. [64]. Briefly, the study involved 50 PLWS attending outpatient services or residential facilities of the Department of Mental Health and Addiction Services—ASST Spedali Civili Brescia (Italy). All participants continued the ongoing pharmacological therapies without any change throughout the study period.

Participants were randomized in a 1:1 ratio to receive active or sham-tDCS. Randomization was provided using a computer-generated random number table without any restriction. Inclusion criteria were (1) adulthood, (2) a diagnosis of schizophrenia according to DSM-5 criteria, and (3) a non-acute phase of the disorder, defined as clinical stability with unchanged pharmacological treatments for at least one month. Exclusion criteria were having (1) organic brain comorbidities, (2) implantable medical devices, (3) history of adverse events to benzodiazepines, (4) recent brain surgery, (5) moderate or severe intellectual disability, (6) active substance use disorders, and (7) pregnancy/breastfeeding.

tDCS protocol (BrainStim stimulator, E.M.S., Bologna, Italy) consisted in a bilateral bipolar-nonbalanced prefrontal electrode montage [66]. According to the EEG 10–20 system, anodal stimulation targeted the left DLPC (F3) and cathodal stimulation the right orbitofrontal region (Fp2), using 5 × 7 cm electrodes covered by saline-soaked sponges. Active-tDCS was delivered as a continuous current at 2 mA for 20 min (+20 s fade-in, +20 s fade-out), whereas sham-tDCS delivered 2 mA current only during fade-in and fade-out periods. Patients received 15 stimulation sessions on weekdays (from Monday to Friday) over a 3-week period.

Clinical assessments included clinician-oriented tools and were conducted at baseline (T0) and after completion of the last tDCS session (T3). All evaluations were performed by trained clinicians with established inter-rater reliability, as documented in prior studies conducted within the same department [64,65]. The Positive and Negative Syndrome Scale (PANSS) [67] was employed to assess clinical symptom severity, providing Positive, Negative, and General Psychopathology subscale scores, as well as a total score. CIAS was evaluated using the Brief Assessment of Cognition in Schizophrenia (BACS) [68] which assesses the main neurocognitive domains impaired in schizophrenia, namely, verbal memory (list learning task), working memory (digit sequencing task), motor speed (token motor task), verbal fluency (verbal fluency task), attention and processing speed (symbol coding task), and executive functions (Tower of London). BACS version 3.1, standardized using normative data from to the Italian population [69] and adjusted for gender and age, was employed. Z-scores were calculated from single tasks’ raw scores and used to derive a composite Global Cognitive Performance score. The Clinical Global Impression (CGI) [70] was used to evaluate the illness severity (CGI-S). Depressive symptoms were assessed using the Calgary Depression Scale for Schizophrenia [71,72], applying a 3-factor solution: depression–hopelessness factor, guilty idea of reference–pathological guilt factor, and early wakening factor.

### 2.2. Statistical Analysis

The original sample was divided according to the smoking status in two subgroups: Smoker patients (SM) and Non-smokers patients (NoSM). Baseline sociodemographic and clinical characteristics comparing SM and NoSM included *χ*^2^-test for categorical variables and independent-sample *t*-test for continuous variables where appropriate. As for the baseline between treatment group (active-tDCS and sham-tDCS) comparisons of demographic and clinical characteristics, *χ*^2^-test for categorical variables and independent-sample *t*-test for continuous variables were used where appropriate.

Given the exploratory and hypothesis-generating nature of these post hoc analyses, parametric statistics were adopted despite deviations from normality in some variables, with the aim of reducing the risk of Type II errors in the context of limited sample sizes. No formal correction for multiple comparisons was applied; therefore, all findings should be interpreted as exploratory rather than confirmatory. Consistent with our previous study, one-way analysis of covariance (ANCOVA) was conducted to assess *between-group* changes over time, using clinical and neuropsychological scores at T3 as dependent variables, treatment group (active-tDCS versus sham-tDCS) as fixed factors and baseline variables (T0) as covariates. When baseline imbalances between treatment arms emerged within a given subgroup, additional covariates were included accordingly. In line with established methodological recommendations, the inclusion of baseline outcome values as covariates represents a standard approach to account for baseline imbalances in RCT and post hoc subgroup analyses [73,74]. Specifically, in the NoSM subgroup, gender and illness duration were included as covariates due to significant baseline differences, whereas no additional covariates beyond baseline outcome values were required in the SM subgroup. IBM^®^SPSS Statistics Version 20 software was used for statistical analyses. Values of *p* < 0.05 were considered significant (2-tails). Cohen’s d was used as an effect size measure and was calculated using an online platform [75,76] with positive scores reflecting positive treatment effects. A d of 0.2 corresponds to a small effect size, a d of 0.5 corresponds to a moderate effect

## 3. Results

### 3.1. Comparison of Baseline Sociodemographic and Clinical Characteristics Between SM and NoSM

The sample included 28 SM and 22 NoSM, distributed among the two treatment groups (Table 1). Within the SM group, 11 patients were allocated to active-tDCS and 17 to sham-tDCS. Conversely, within the NoSM group, 14 patients were allocated to active-tDCS and 8 to sham-tDCS. Although the distribution of patients across treatment groups and smoking status was overall balanced (*p* = 0.087), it should be noted that within the sham-tDCS group, there were more than twice as many smokers (n = 17) as non-smokers (n = 8).

According to the smoking status, baseline sociodemographic and clinical characteristics are summarized in Table 2; as for sociodemographic variables, greater education levels were found in the NoM sample (*p* = 0.01), while no other differences were observed between the SM and NoSM groups.

As for clinical variables, no difference between the SM and NoSM groups emerged for the PANSS, CGI-S and CDSS scores (all, *p* > 0.05) at baseline. Conversely, the SM group showed greater cognitive impairment at the BACS for the following domains: verbal memory (*p* = 0.03), motor speed (*p* = 0.01) and working memory (*p* = 0.04), as well as marginal lower BACS composite score (*p* = 0.05).

### 3.2. tDCS Effects on Clinical Outcomes in the SM Group

Across the SM group, 11 patients were allocated to the active-tDCS arm and 17 patients to the sham-tDCS. Considering treatment allocation to either active or sham-tDCS, SM did not differ in any sociodemographic and clinical characteristics at baseline, except for higher PANSS-Negative subscale (*p* = 0.02), PANSS General Psychopathology (*p* = 0.03), and PANSS total scores (*p* = 0.03) in the active-tDCS group (Supplementary Materials Table S1).

Despite these baseline differences observed in the SM group, no additional adjustments were deemed necessary in the subsequent between-groups analyses, as baseline PANSS values had already been included as covariates in the ANCOVA models (Table 3). The ANCOVA demonstrated significantly greater reductions in the PANSS-Negative subscale (*p <* 0.001, d = 1.62) and PANSS total score (*p* = 0.003, d = 0.87) in the active-tDCS group compared to sham-tDCS, supporting the effectiveness and superiority of the active intervention in improving these outcomes. A trend toward significance was observed for the PANSS General Psychopathology subscale (*p* = 0.05, d = 0.71). With respect to CGI-S, active-tDCS outperformed sham-tDCS in improving illness severity score (*p* = 0.003, d = 0.63). Regarding CIAS, the ANCOVA analyses did not identify any significant between-group improvement in any of the cognitive domains assessed by the BACS (all, *p* > 0.05). Likewise, no significant between-group differences over time were observed in any CDSS scores, indicating no differential effects on depressive symptoms (all *p* > 0.05)

### 3.3. tDCS Effects on Clinical Outcomes in the NoSM Group

In the NoSM group, 14 patients received active-tDCS and 8 patients received sham-tDCS. When considering treatment allocation to active or sham-tDCS, the NoSM group did not differ in any sociodemographic and clinical characteristics at baseline, except for gender distribution (*p* = 0.005) and illness duration (*p* = 0.007), which was longer in the sham-tDCS group (Supplementary Materials Table S2). Moreover, if compared with active-tDCS group, patients allocated to sham-tDCS group showed lower cognitive scores in the following domains: motor speed (*p* = 0.03), verbal memory (*p* = 0.001), verbal fluency (*p* = 0.03), and lower BACS composite scores (*p* = 0.02 (Table S2). Despite these differences, no additional adjustments were required in the subsequent ANCOVA, as the baseline cognitive values were already included as covariates. Conversely, as gender and illness duration could negatively impact the changes over time of clinical outcomes, these variables were included in the following ANCOVA as covariates.

ANCOVA analyses (Table 4) demonstrated the superiority of the active-tDCS compared with sham-tDCS in improving the PANSS-Negative subscale (*p* = 0.001, d = 1.11) and the PANSS total score (*p* = 0.001, d = 0.72). Trends toward significance were observed for the PANSS General Psychopathology subscale (*p* = 0.06, d = 0.53) and for CGI-S (*p* = 0.06, d = 0.74). With regard to CIAS, ANCOVA revealed significant improvements in favor of active-tDCS in the working memory (*p* = 0.04, d = 0.53) and verbal fluency (*p* = 0.04, d = 0.55) domains. Regarding depressive symptoms, ANCOVA revealed significant improvements favoring active-tDCS exclusively for the CDSS Guilt–Self-depreciation factor (*p* = 0.009, d = 0.08).

## 4. Discussion

The present study aimed to investigate the potential moderating role of cigarette smoking, conceptualized as a modifiable individual factor, on the efficacy of a bilateral bipolar non-balanced prefrontal tDCS protocol in improving NS and CIAS in schizophrenia. Moreover, considering theoretical accounts suggesting that SM exhibited more pronounced cognitive impairment than NoSM, we also examined whether, at baseline, SM showed poorer cognitive performance compared with NoSM.

We first observed that compared with NoSM, SM showed significantly poorer performance across multiple cognitive tasks domains, including verbal memory, motor speed and working memory, as well as lower BACS composite score. These findings are consistent with previous evidence indicating that PLWS who smoke exhibit greater cognitive impairments compared with non-smokers [32,33,35,36,37,38], indirectly supporting the notion that the long-term use of cigarettes is associated with detrimental long-term effects not only on physical health but also on cognition in schizophrenia. Moreover, these findings implicitly confirm and are consistent with the self-medication theory that suggested that PLWS may use cigarette smoking as a compensatory strategy to alleviate CIAS and NS, given the acute effects of nicotine on dopamine release in the prefrontal cortex [30,32,77,78].

Subsequently, we examined the differential effects of tDCS in SM and NoSM on psychopathological and cognitive outcomes. Within the SM group, we found that, compared with sham treatment, active-tDCS produced significant improvements in the PANSS-Negative subscale and PANSS total score, which were both associated with large effect sizes, whereas only a trend-level effect was observed for the PANSS General Psychopathology subscale. Moreover, clinical severity significantly improved following active-tDCS with a moderate effect size. With respect to cognitive outcomes, no significant improvements following tDCS were observed in the SM group across any cognitive domains assessed by the BACS. Similarly, depressive symptoms in SM were not significantly affected by tDCS, with the exception of a trend-level improvement in the CDSS total score.

On the other hand, in the NoSM group, we found that, compared with sham treatment, active-tDCS produced significant improvements in the PANSS-Negative subscale (with a large effect size), the PANSS total score (with a moderate-to-large effect size), and in a specific dimension of depressive symptoms, namely the Guilt–Self-depreciation factor (with a large effect size). Conversely, the PANSS General Psychopathology subscale and clinical severity exhibited improvements only approaching statistical significance. With regard to baseline cognitive functioning in the NoSM group, patients assigned to sham-tDCS exhibited poorer cognitive performance than those receiving active-tDCS, particularly in motor speed, verbal learning and memory, and verbal fluency, as well as in the BACS composite score. Although the scientific literature did not provide consistent evidence for the progressive worsening of CIAS throughout the lifespan [24,79,80], we can hypothesize that the greater cognitive impairments may be partly explained by the longer illness duration observed in patients allocated to sham-tDCS. Nevertheless, at the study completion, active-tDCS outperformed sham treatment in enhancing working memory and verbal fluency, with both effects reaching a moderate magnitude.

Overall, these findings further support the specific efficacy of bilateral bipolar non-balanced prefrontal tDCS in improving negative—but not positive—symptoms in schizophrenia, in line with previous studies [64,81,82] and consistent with a recent meta-analysis [43]. Importantly, they also provide preliminary evidence that NS improvements occurred independently of cigarette smoking status. Moreover, NS improvements in both SM and NoSM were associated with large effect sizes. With regard to CIAS, while no meaningful changes in cognitive outcome were observed in SM, active-tDCS was superior to sham-tDCS in improving working memory and verbal fluency domains, with moderate effect sizes, exclusively in the NoSM group. Although these findings are preliminary and based on small sample sizes, this study suggests, for the first time, that cigarette smoking may represent an individual factor limiting the effectiveness of this tDCS protocol in mitigating CIAS. At the same time, our findings are in line with meta-analytic evidence demonstrating significant effects of tDCS on working memory [42,56]. Furthermore, they extend the existing evidence of tDCS effects on verbal fluency in schizophrenia, given that only a limited number of studies have reported significant improvements in this cognitive domain following either prefrontal [81,82,83] or fronto-temporal tDCS [84]. Nevertheless, these findings should be interpreted cautiously, as they derive from exploratory subgroup analyses without correction for multiple testing. Although effect sizes were in the moderate range, the present results cannot be considered definitive and require replication in adequately powered prospective studies.

With regard to other clinical outcomes, clinical severity significantly improved following active-tDCS only in the SM group, whereas in the NoSM, only a trend-level improvement was observed on the CGI-S. These findings, which are not fully consistent with PANSS scores changes observed in both SM and NoSM, could be attributable to the small sample size of the NoSM group and should not be solely ascribed to the smoking status. Similarly, improvements in specific depressive symptoms were observed only in the NoSM group, whereas active-tDCS produced only a trend-level improvement in the CDSS total score in the SM group. Given that previous tDCS studies using the same electrode montage reported improvements in depressive symptoms in schizophrenia [65,70], the discrepancies observed in our results are more likely related to an increased risk of Type II errors rather than to a direct effect of smoking status.

Together with the findings reported by Brunelin et al. [62], our results suggest that cigarette smoking represents a modifiable individual factor that may negatively influence the clinical outcomes of tDCS in schizophrenia, particularly with respect to CIAS. Similar findings arise from a recent meta-analysis on tDCS in improving NS in which tobacco smoking was identified as a significant moderator, though it did not reach significance within either the active or sham-tDCS [57]. This pattern suggested an interaction-driven moderation effect rather than a direct within-group effect. Notably, the authors also reported a trend toward significance in the active-tDCS group, indicating that smoking status was associated with worse clinical outcomes, a pattern not observed in the sham-tDCS group [57]. Nevertheless, our suggestions warrant cautious interpretation, as other studies have reported favorable effects of smoking on neuroplasticity in schizophrenia. For instance, Strube et al. reported that tobacco smoking induced a restitution of impaired LTD-like neuroplasticity in SM and abolished LTD-like plasticity in NoSM patients following 8 min cathodal-tDCS over the left primary motor cortex [85]. Consistent with the self-medication theory, these findings suggested that nicotine intake might improve NS and CIAS by stabilizing the impaired balance between excitatory and inhibitory neurons through complex interactions on cortical plasticity and GABAergic/cholinergic transmission [85]. However, these results also highlight that the net effect of tobacco smoking on tDCS effectiveness may be influenced by unmeasured confounding variables intrinsic to cigarette smoking, which Guiomar et al. described as “metabolic factors” [57].

From our perspective, these “metabolic factors” related to tobacco smoking may be explained by considering that cigarettes contain several neurotoxic molecules, including carbon monoxide, acrolein, acetaldehyde, hydrogen cyanide, acetaldehyde, formaldehyde, ammonia, cresol, catechol, hydroquinone, methyl–ethyl–ketone, nitric oxide, phenol, styrene, toluene, butane, as well as various heavy metals (e.g., arsenic, cadmium, chromium, lead, polonium, and nickel) [60,61]. A suggestion is that chronic cigarette smoking could be responsible for *neurotoxic milieu*—characterized by increased oxidative stress and glutamate neurotoxicity, inflammation, and atherosclerosis—thereby contributing to progressive neurodegeneration and cognitive deterioration [30,60,61]. Importantly, this interpretation should be regarded as hypothesis-generating, based on indirect evidence investigating the effects of chronic cigarette smoking in schizophrenia [38,60,61,78].

Within this framework, independently of nicotine’s acute action, we can hypothesize that net effect of cigarette smoking on brain structure and cognitive functioning in schizophrenia may be extremely complex and detrimental. This notion is indirectly supported by structural neuroimaging studies linking smoking status to reduced gray matter volumes, particularly within prefrontal regions [86,87,88,89]. Accordingly, we hypothesize that a *neurotoxic milieu* may interfere with the neuroprotective effects of tDCS (i.e., anti-inflammatory, pro-angiogenic, and anti-apoptotic actions), potentially contributing to the lack of cognitive improvement observed in SM following tDCS. However, this proposed mechanism has not been empirically demonstrated and should be interpreted with caution. Future studies integrating biological markers (e.g., indices of oxidative stress and inflammation, as well as neuroimaging measures of cortical integrity) are required to directly test this hypothesis and to clarify the biological mechanisms underlying the interaction between smoking status and tDCS responsiveness.

On the other hand, while the *neurotoxic milieu* hypothesis may help explain the differential effect of smoking on CIAS, we observed that cigarette smoking did not impact tDCS effects on NS, as both SM and NoSM groups showed significant improvements in this clinical dimension. Given the shared neurobiological substrates underlying CIAS and NS [1,2,12], the absence of specific effects of cigarette smoking on NS remains difficult to interpret, particularly in light of the findings reported by Guiomar et al. [57]. One possible explanation is that the available scientific evidence on the relationship between NS severity and cigarette smoking status remains inconclusive [30,90,91]. Therefore, further studies are needed to confirm and extend our preliminary observations.

This study has several limitations. The most important is that the results were derived from post hoc secondary analyses from an RCT that was not specifically designed to evaluate the effect of smoking status as a possible individual moderator on clinical outcomes. As a consequence, findings regarding the interaction between smoking status and tDCS effectiveness must be interpreted with caution as they are explorative and require further replication in adequately powered prospective studies. Another important limitation concerns the absence of a formal correction for multiple comparisons across cognitive domains and clinical endpoints, which increases the risk of false-positive findings. Accordingly, cognitive outcomes should be regarded as exploratory and interpreted within a hypothesis-generating framework. Although the original study enrolled 50 patients, stratification by smoking status and treatment allocation resulted in small sample sizes, despite an overall balanced distribution. This likely reduced the statistical power of the analyses, increasing the risk of Type II error and limiting the ability to detect small-to-moderate effect sizes. Another limitation concerns the incomplete characterization of smoking behaviors. As the investigation of tDCS effects on cigarette consumption or craving was beyond the scope of the present study, the smoking status was dichotomously operationalized without a quantitative assessment of tobacco exposure. Moreover, detailed measures of smoking intensity and nicotine dependence (including duration of exposure, age at smoking onset) were not systematically collected in the original RCT because the study was not specifically designed to systematically investigate smoking-related variables. This methodological simplification precluded an accurate examination of potential dose–response relationship and did not allow a clear distinction between the acute effects of nicotine and the neurotoxic consequences of chronic cigarette smoking. Therefore, further perspective studies that include standardized quantitative assessments of smoking behaviors are needed to better characterize smoking exposure and its interaction with tDCS-induced neuroplasticity. Another limitation concerns the intrinsic clinical heterogeneity of the sample, which is typical of studies conducted in naturalistic settings. Although the SM and NoSM groups were largely comparable across most sociodemographic and pharmacological variables, some baseline differences emerged when treatment allocation was considered. Despite being statistically controlled for using ANCOVA, these differences may nonetheless have influenced the clinical outcomes. Considering the study design, the lack of a follow-up period limits the possibility to evaluate the durability of clinical changes and does not allow evaluations of whether the smoking status may influence tDCS effects over the medium-to-long term. Similarly, the evaluation of possible differential long-term trajectories between SM and NoSM remains unknown. Longitudinal studies with extended follow-up periods are therefore warranted to determine whether smoking status influences not only the acute effects of tDCS but also the long-term sustainability of clinical benefits. Finally, another important limitation is the absence of biological markers to clarify the mechanism underlying the relationship between the effects of smoking status and cognitive outcome across the SM and NoSM groups. As we did not collect any oxidative stress, inflammatory pathways, or neuroimaging indices, the hypothesis that chronic cigarette smoking is associated with a *neurotoxic milieu* responsible for worse cognitive outcomes in the SM group remains purely speculative as no causal or mechanistic inferences can be definitively drawn from our data.

## 5. Conclusions

This study evaluated, though post hoc analyses, whether the smoking status may impact the effects of a bilateral bipolar non-balanced prefrontal tDCS protocol in improving NS and CIAS in schizophrenia. In this study, given the well-known role of cigarette smoking in influencing CIAS, smoking status was examined as a modifiable individual factor with the aim to reduce heterogeneity in NIBS studies linked to sample characteristics. Our findings indicate that cigarette smoking was not associated with differential tDCS effects in improving NS. In contrast, improvements in specific cognitive domains—namely working memory and verbal fluency—were observed only in the NoSM group. Importantly, these findings should be interpreted as exploratory rather than confirmatory, given the post hoc nature of the analyses, the limited statistical power of subgroup comparisons, and the presence of multiple outcomes. Accordingly, causal inferences cannot be drawn. The dichotomous classification of smoking status and the incomplete characterization of smoking-related behaviors further preclude dose–response interpretations and may mask heterogeneity within the smoker subgroup. Alternative explanations, including baseline cognitive differences, residual confounding despite statistical adjustment, unmeasured medication- or lifestyle-related factors associated with smoking status, and findings potentially influenced by an increased risk of Type I error associated with multiple testing, cannot be excluded.

Within this context, the hypothesis of a smoking-related *neurotoxic milieu*—linked to chronic exposure to neurotoxic molecules constituent of cigarette smoking—that may potentially counteract tDCS-induced neuroplastic effects must be regarded as speculative and hypothesis-generating, rather than as an empirically demonstrated mechanism.

Overall, while these findings contribute to refining our understanding of individual factors that may influence tDCS effectiveness in schizophrenia, future prospective studies with larger samples, detailed quantitative characterization of tobacco exposure, and the integration of neurobiological markers will be necessary to confirm and further elucidate these preliminary observations.

## Figures and Tables

**Table 1:** Distribution of Smoker and Non-smoker patients across the active and sham-tDCS groups.

Sample	Treatment Group			*χ^2^*-Test	*p*-Value
	Active-tDCS (n, %)	Sham-tDCS (n, %)	Total (n, %)		
**Smoker**	11 (22%)	17 (34%)	28 (56%)	2.922	0.087
**Non-smoker**	14 (28%)	8 (16%)	22 (44%)		
**Total**	25 (50%)	25 (50%)	50 (100%)		

**Table 2:** Comparison of baseline sociodemographic and clinical characteristics comparing Smoker and Non-smoker patients.

Variable	Sample		Tests *(χ*^2^-Test, *t*-Test)	*p*-Value
	Smoker (n = 28)	Non-Smoker (n = 22)		
**Sociodemographic**				
Gender (male/female)	23/5	16/6	0.636	0.42
Age (years)	40.7 ± 12.9	44.8 ± 12.2	−1.131	0.26
Education (years)	10.61 ± 2.51	12.77 ± 3.24	−2.665	**0.01**
Ethnicity (Caucasian)	23	18	1.41	0.49
Handedness (right/left)	24/4	21/1	1.299	0.25
Age at onset (years)	27.18 ± 8.30	25.41 ± 9.70	0.70	0.50
Illness duration (years)	14.82 ± 9.35	16.18 ± 9.66	−0.503	0.62
**Pharmacotherapies**				
Chlorpromazine equivalents	623.495 ± 399.410	476.388 ± 276.507	1.471	0.15
Clozapine	4	3	0.004	0.95
LAI	16	9	1.299	0.25
Mood stabilizers	5	4	0.001	0.97
Benzodiazepines	18	14	0.002	0.96
Anticholinergics	9	9	0.411	0.52
Antidepressants	4	5	0.595	0.44
**Psychopathology**				
**PANSS**				
Positive subscale	16.68 ± 3.63	15.41 ± 5.21	1.015	0.32
Negative subscale	26.21 ± 3.24	25.09 ± 4.42	1.040	0.30
General Psychopathology subscale	39.71 ± 6.64	36.14 ± 7.59	1.777	0.08
PANSS Total score	82.61 ± 11.27	76.64 ± 16.00	1.55	0.13
**CGI-S**	4.50 ± 0.75	4.41 ± 0.67	0.450	0.65
**BACS**				
Token motor	−4.36 ± 1.30	−3.43 ± 1.35	−2.467	**0.02**
Verbal memory	−2.43 ± 0.86	−1.70 ± 1.44	−2.238	**0.03**
Digit sequencing	−1.74 ± 1.07	−1.09 ± 1.04	−2.163	**0.04**
Symbol coding	−2.67 ± 1.30	−2.19 ± 1.55	−1.187	0.24
Verbal fluency	−1.59 ± 0.73	−1.56 ± 1.06	−0.094	0.92
Tower of London	−1.12 ± 1.63	−0.76 ± 1.12	−0.880	0.38
Composite Score	−2.28 ± 0.81	−1.76 ± 1.04	−1.999	**0.05**
**CDSS**				
Depression–Hopelessness factor	4.500 ± 3.383	4.364 ± 2.888	0.151	0.88
Guilt–Self-depreciation factor	1.393 ± 1.474	1.546 ± 1.738	−3.336	0.74
Early wakening factor	0.39 ± 0.69	0.73 ± 1.03	−1.374	0.18
Total score	6.46 ± 4.57	6.64 ± 4.28	−0.136	0.89

BACS: Brief Assessment Cognition Schizophrenia; CDSS: Calgary Depression Scale for Schizophrenia; CGI-S: Clinical Global Impression rating scale—Severity index; LAI: Long-Acting Injectable; n: number; PANSS: Positive and Negative Syndrome Scale. The PANSS, CGI-S, BACS and CDSS scores are expressed as “means” ± “standard deviations”. Regarding BACS, reported scores are expressed as “z-scores” derived from raw scores of the individual tasks. For continuous sociodemographic variables (i.e., age, years of education, age at onset, duration of illness, and chlorpromazine equivalents), data are presented as “means” ± “standard deviations”. For the remaining categorical sociodemographic variables, data are reported as raw frequencies. Bold formatting is used to highlight statistically significant *p*-values.

**Table 3:** tDCS effects on clinical outcomes in Smoker patients.

Variable	tDCS Group	Pre-Treatment (Mean ± SD)	Post-Treatment (Mean ± SD)	ANCOVA		
				F	*p*-Value	Cohen’s d
**PANSS**						
Positive subscale	Active	17.27 ± 2.61	16.64 ± 3.47	0.001	0.97	0.05
	Sham	16.29 ± 4.20	15.65 ± 4.27			
Negative subscale	Active	27.91 ± 3.18	22.55 ± 2.54	72.42	**<0.001**	1.62
	Sham	25.12 ± 2.85	24.76 ± 3.21			
General Psychopathology subscale	Active	43.00 ± 6.13	37.36 ± 3.50	4.049	0.05	0.71
	Sham	37.59 ± 6.22	36.41 ± 7.01			
PANSS Total score	Active	88.18 ± 8.10	76.55 ± 4.89	10.585	**0.003**	0.87
	Sham	79.00 ± 11.75	76.82 ± 12.90			
**CGI-S**	Active	4.55 ± 0.93	4.00 ± 0.00	5.434	**0.03**	0.63
	Sham	4.47 ± 0.62	4.41 ± 0.71			
**BACS**						
Token motor	Active	−3.98 ± 1.53	−3.89 ± 1.12	0.130	0.72	0.27
	Sham	−4.60 ± 1.11	−4.14 ± 1.35			
Word recall	Active	−2.52 ± 0.79	−1.79 ± 1.04	0.555	0.46	0.22
	Sham	−2.37 ± 0.93	−1.44 ± 1.04			
Digit sequencing	Active	−1.63 ± 0.99	−1.27 ± 1.10	0.852	0.36	0.20
	Sham	−1.81 ± 1.13	−1.68 ± 1.02			
Symbol coding	Active	−2.54 ± 1.16	−2.32 ± 1.15	0.418	0.52	0.07
	Sham	−2.76 ± 1.42	−2.62 ± 1.11			
Verbal fluency	Active	−1.41 ± 0.71	−1.21 ± 0.57	1.831	0.19	0.21
	Sham	−1.70 ± 0.75	−1.66 ± 0.72			
Tower of London	Active	−1.33 ± 1.86	−0.50 ± 1.82	0.833	0.37	0.28
	Sham	−0.98 ± 1.52	−0.63 ± 1.09			
Composite score	Active	−2.18 ± 0.85	−1.83 ± 0.84	0.178	0.67	0.04
	Sham	−2.35 ± 0.81	−2.03 ± 0.70			
**CDSS**						
Total score	Active	8.29 ± 5.46	3.36 ± 4.88	3.831	0.06	0.86
	Sham	5.29 ± 3.60	4.29 ± 3.89			
Depression–Hopelessness factor	Active	6.00 ± 3.74	2.45 ± 3.17	3.411	0.08	0.91
	Sham	3.53 ± 2.83	3.00 ± 3.37			
Guilt–Self-depreciation factor	Active	2.00 ± 1.84	0.72 ± 1.27	2.95	0.09	0.80
	Sham	1.00 ± 1.06	0.82 ± 0.81			
Early wakening factor	Active	0.27 ± 0.65	0.18 ± 0.60	0.12	0.73	0.12
	Sham	0.47 ± 0.72	0.47 ± 0.71			

ANCOVA: Analysis of Covariance; BACS: Brief Assessment Cognition Schizophrenia; CDSS: Calgary Depression Scale for Schizophrenia; CGI: Clinical Global Impression; PANSS: Positive and Negative Syndrome Scale; SD: standard deviations; tDCS = transcranial Direct Current Stimulation. In this table, the PANSS, CGI-S, BACS and CDSS scores are expressed as “means” ± “standard deviations”. For BACS, reported scores are expressed as “z-scores” derived from raw scores of the individual tasks. This table represents findings from the one-way analyses of covariance (ANCOVA) that were conducted to assess between-group changes over time in the SM group, using clinical and neuropsychological scores at T3 as dependent variables, treatment group (active-tDCS versus sham-tDCS) as fixed factors and baseline variables (T0) as covariates. Despite differences observed between active and sham-tDCS groups on baseline values of PANSS-Negative subscale, PANSS General Psychopathology and PANSS total scores, no additional adjustments were deemed necessary in the subsequent analyses as all baseline PANSS values had already been included as covariates in the ANCOVA models. Bold formatting is used to highlight statistically significant *p*-values.

**Table 4:** tDCS Effects on clinical outcomes in Non-smoker patients.

Variable	tDCS Group	Pre-Treatment (Mean ± SD)	Post-Treatment (T3) (Mean ± SD)	ANCOVA		
				F	*p*-Value	Cohen’s d
**PANSS**						
Positive subscale	Active	15.07 ± 5.44	13.79 ± 4.89	3.734	0.07	0.16
	Sham	16.00 ± 5.07	15.63 ± 5.13			
Negative subscale	Active	24.86 ± 4.99	18.50 ± 5.24	15.261	**0.001**	1.11
	Sham	25.50 ± 3.46	24.38 ± 3.11			
General Psychopathology subscale	Active	35.71 ± 7.95	30.43 ± 6.73	4.183	0.06	0.53
	Sham	36.88 ± 7.38	34.87 ± 6.03			
PANSS Total score	Active	75.64 ± 17.10	62.71 ± 14.13	14.941	**0.001**	0.72
	Sham	78.38 ± 14.79	74.88 ± 12.56			
**CGI-S**	Active	4.29 ± 0.73	3.79 ± 0.58	4.106	0.06	0.74
	Sham	4.63 ± 0.52	4.50 ± 0.54			
**BACS**						
Token motor	Active	−2.95 ± 1.40	−2.61 ± 2.06	0.341	0.57	0.26
	Sham	−4.25 ± 0.79	−3.58 ± 1.61			
Word recall	Active	−0.99 ± 1.21	−0.88 ± 1.24	0.360	0.56	0.24
	Sham	−2.92 ± 0.90	−2.63 ± 1.01			
Digit sequencing	Active	−0.80 ± 1.09	−0.49 ± 1.14	4.886	**0.04**	0.53
	Sham	−1.60 ± 0.75	−1.84 ± 0.46			
Symbol coding	Active	−1.95 ± 1.70	−1.68 ± 1.87	0.188	0.67	0.34
	Sham	−2.62 ± 1.25	−2.91 ± 1.28			
Verbal fluency	Active	−1.21 ± 1.09	−1.07 ± 1.41	4.721	**0.04**	0.55
	Sham	−2.18 ± 0.65	−1.63 ± 0.80			
Tower of London	Active	−0.52 ± 1.07	−0.32 ± 1.35	2.081	0.17	0.09
	Sham	−1.18 ± 1.15	−1.09 ± 1.69			
Composite score	Active	−1.37 ± 1.09	−1.17 ± 1.24	0.121	0.73	0.05
	Sham	−2.43 ± 0.48	−2.28 ± 0.44			
**CDSS**						
Total score	Active	6.36 ± 3.87	2.21 ± 1.53	3.282	0.08	0.61
	Sham	7.13 ± 5.16	5.75 ± 4.50			
Depression–Hopelessness factor	Active	4.36 ± 2.47	0.93 ± 1.27	2.403	0.14	0.80
	Sham	4.38 ± 3.70	3.38 ± 2.92			
Guilt–Self-depreciation factor	Active	1.29 ± 1.27	0.64 ± 0.63	8.800	**0.009**	0.08
	Sham	2.00 ± 2.39	1.50 ± 1.41			
Early wakening factor	Active	0.64 ± 0.84	0.64 ± 0.74	1.39	0.25	0.009
	Sham	0.88 ± 1.36	0.87 ± 1.35			

ANCOVA: Analysis of Covariance; BACS: Brief Assessment Cognition Schizophrenia; CDSS: Calgary Depression Scale for Schizophrenia; CGI: Clinical Global Impression; PANSS: Positive and Negative Syndrome Scale; SD: standard deviations; tDCS = transcranial Direct Current Stimulation. PANSS, CGI-S, BACS and CDSS scores are expressed as “means” ± “standard deviations”. For BACS, reported scores are expressed as “z-scores” derived from raw scores of the individual tasks. This table represents findings from the one-way analyses of covariance (ANCOVA) that were conducted to assess between-group changes over time in the NoSM group, using clinical and neuropsychological scores at T3 as dependent variables, treatment group (active-tDCS versus sham-tDCS) as fixed factors and baseline variables (T0) as covariates. Given the differences observed between active and sham-tDCS groups on baseline sociodemographic characteristics (gender and illness duration), all the ANCOVA were adjusted considering these two sociodemographic variables as covariates. Conversely, despite baseline differences across treatment groups for some cognitive scores (token motor, verbal memory, verbal fluency tasks and BACS composite score), no additional adjustments were required in the subsequent ANCOVA as the baseline cognitive values were already included as covariates in the analyses. Bold formatting is used to highlight statistically significant *p*-values.

## Data Availability

The data presented in this study are available on request from the corresponding author due to privacy and legal reasons.

## Supplementary Materials

The following supporting information can be downloaded at https://www.mdpi.com/article/10.3390/brainsci16020186/s1. Table S1. Baseline sociodemographic and clinical characteristics across treatment groups (active-tDCS vs sham-tDCS) in Smoker patients. Table S2. Baseline sociodemographic and clinical characteristics across treatment groups (active-tDCS vs. sham-tDCS) in Non-Smoker patients.

## Abbreviations

ANCOVA	analyses of covariance
AVH	auditory verbal hallucinations
BACS	Brief Assessment of Cognition in Schizophrenia
CDSS	Calgary Depression Scale for Schizophrenia total score
CGI	Clinical Global Impression
CIAS	Cognitive Impairment Associated with Schizophrenia
DLPFC	Dorsolateral Prefrontal Cortex
EEG	Electroencephalography
FGAs	first-generation antipsychotics
GABA	γ-Aminobutyric acid
LAI	*Long Acting Injectable*
LTD	long-term depression
LTP	long-term potentiationNIBS—Non-Invasive Brain Stimulation
NMDA	N-methyl-D-aspartate
NS	Negative symptoms
NoSM	Non-Smoker patients
PANSS	Positive and Negative Symptoms Scale
PLWS	people living with schizophrenia
RCT	randomized controlled trial
rTMS	repetitive transcranial Magnetic Stimulation
SGAs	second-generation antipsychotics
SM	Smoker patients
T	timepoint
tDCS	transcranial Direct Current Stimulation
