# Supplementary material for: Moderating Role of Cigarette Smoking on the Efficacy of tDCS in the Treatment of Negative and Cognitive Symptoms of Schizophrenia: Results from a Randomized Clinical Trial
Source: Brain Sci. 2026 Feb 3;16(2):186. doi: 10.3390/brainsci16020186 (PMC12938019; doi:10.3390/brainsci16020186)
Supplement: Supplementary file 1 [file brainsci-16-00186-s001.zip › brainsci-4089936-supplementary.pdf]

Table S1. Baseline sociodemographic and clinical characteristics across treatment groups (active-tDCS vs sham-tDCS) in Smoker patients.

Variable	Sample		Test (χ^2/*t*-test)	*p*-value
	**active-tDCS** (*n* = 11)	**sham-tDCS** (*n* = 17)		
**Sociodemographic**				
Gender (male/female)	10/1	13/4	*0*.950	0.33
Age (years, mean ± SD)	43.73 ± 14.17	38.76 ± 11.99	0.996	0.33
Education (years, mean ± SD)	9.45 ± 2.16	11.35 ± 2.50	-2.066	0.05
Ethnicity (Caucasian)	8	15	1.09	0.29
Handedness (right/left)	10/1	14/3	0.399	0.53
Age at onset (years, mean ± SD)	28.18 ± 8.45	26.53 ± 8.53	0.502	0.62
Duration of illness (mean ± SD)	15.55 ± 9.98	14.35 ± 9.19	0.324	0.75
**Pharmacotherapies**				
Chlorpromazine equivalents (mean ± SD)	684.65 ± 392.77	583.92 ± 410.56	0.645	0.52
Clozapine (n)	2	2	0.22	0.63
LAI (n)	5	11	1.011	0.31
Mood stabilizers (n)	1	4	0.949	0.33
Benzodiazepines (n)	8	10	0.562	0.45
Anticholinergics (n)	5	4	1.472	0.22
Antidepressants (n)	2	2	0.225	0.63
**Psychopathology**				
**PANSS**				
Positive subscale (mean ± SD)	17.27 ± 2.61	16.29 ± 4.20	0.690	0.50
Negative subscale (mean ± SD)	27.91 ± 3.18	25.12 ± 2.85	2.422	**0.02**
General Psychopathology subscale (mean ± SD)	43.00 ± 6.13	37.59 ± 6.22	2.262	**0.03**
PANSS Total score (mean ± SD)	88.18 ± 8.10	79.00 ± 11.75	2.261	**0.03**
**CGI-S** (mean ± SD)	4.55 ± 0.93	4.47 ± 0.62	0.255	0.80
**BACS**				
Token motor (z-score, mean ± SD)	-3.98 ± 1.53	-4.60 ± 1.11	1.229	0.23
Verbal memory (z-score, mean ± SD)	-2.52 ± 0.79	-2.37 ± 0.93	-0.437	0.66
Digit sequencing (z-score, mean ± SD)	-1.63 ± 0.997	-1.81 ± 1.13	0.430	0.67
Symbol coding (z-score, mean ± SD)	-2.54 ± 1.16	-2.76 ± 1.42	0.425	0.67
Verbal fluency (z-score, mean ± SD)	-1.41 ± 0.71	-1.70 ± 0.75	1.003	0.32
Tower of London (z-score, mean ± SD)	-1.33 ± 1.86	-0.98 ± 1.52	-0.549	0.60
Composite Score (z-score, mean ± SD)	-2.18 ± 0.85	-2.35 ± 0.81	0.516	0.61
**CDSS**				
Depression Hopelessness factor (mean ± SD)	6.00 ± 3.74	3.53 ± 2.83	0.199	0.06
Guilt-Self depreciation factor (mean ± SD)	2.00 ± 1.84	1.00 ± 1.06	1.827	0.08
Early wakening factor (mean ± SD)	0.27 ± 0.65	0.47 ± 0.72	-0.740	0.46
Total score (mean ± SD)	8.29 ± 5.46	5.29 ± 3.60	1.74	0.90

BACS: Brief Assessment Cognition Schizophrenia; CDSS: Calgary Depression Scale for Schizophrenia; CGI: Clinical Global Impression rating scales; LAI: Long Acting Injectable; n: number; PANSS: Positive and Negative Syndrome Scale, SD: standard deviations; tDCS: transcranial Direct Current Stimulation.

Table S2. Baseline sociodemographic and clinical characteristics across treatment groups (active-tDCS vs sham-tDCS) in Non-Smoker patients.

Variable	Sample		Test (χ^2/*t*-test)	*p*-value
	**active-tDCS** (*n* = 14)	**sham-tDCS** (*n* = 8)		
**Sociodemographic**				
Gender (male/female)	13/1	3/5	7.865	**0.005**
Age (years, mean ± SD)	42.79 ± 12.30	48.25 ± 12.10	-1.008	0.32
Education (years, mean ± SD)	13.14 ± 2.57	12.13 ± 4.29	0.701	0.49
Ethnicity (Caucasian)	11	7	6.33	0.73
Handedness (right/left)	13/1	8/0	0.599	0.44
Age at onset (years, mean ± SD)	26.57 ± 9.01	23.38 ± 11.14	0.735	0.47
Duration of illness (mean ± SD)	12.21 ± 6.36	23.13 ± 10.88	-2.991	**0.007**
**Pharmacotherapies**				
Chlorpromazine equivalents (mean ± SD)	428.84 ± 235.12	559.60 ± 338.04	-1.071	0.30
Clozapine (n)	1 (7.1%)	2 (25.0%)	1.378	0.24
LAI (n)	6 (42.9%)	3 (37.5%)	0.060	0.80
Mood stabilizers (n)	4 (28.6%)	0 (0.0%)	2.794	0.09
Benzodiazepines (n)	9 (64.3%)	5 (62.5%)	0.007	0.93
Anticholinergics (n)	6 (42.9%)	3 (37.5%)	0.060	0.81
Antidepressants (n)	3 (21.43%)	2 (25%)	0.037	0.84
**Psychopathology**				
**PANSS**				
Positive subscale (mean ± SD)	15.07 ± 5.44	16.00 ± 5.07	-0.394	0.70
Negative subscale (mean ± SD)	24.86 ± 4.99	25.50 ± 3.46	-0.321	0.75
General Psychopathology subscale (mean ± SD)	35.71 ± 7.95	36.88 ± 7.38	-0.338	0.74
PANSS Total score (mean ± SD)	75.64 ± 17.10	78.38 ± 14.79	-0.378	0.71
**CGI-S** (mean ± SD)	4.29 ± 0.73	4.63 ± 0.52	-1.159	0.26
**BACS**				
Token motor (z-score, mean ± SD)	-2.95 ± 1.40	-4.25 ± 0.79	2.411	**0.03**
Verbal memory (z-score, mean ± SD)	-0.99 ± 1.21	-2.92 ± 0.90	3.922	**0.001**
Digit sequencing (z-score, mean ± SD)	-0.80 ± 1.09	-1.60 ± 0.75	1.809	0.08
Symbol coding (z-score, mean ± SD)	-1.95 ± 1.70	-2.62 ± 1.25	0.971	0.34
Verbal fluency (z-score, mean ± SD)	-1.21 ± 1.09	-2.18 ± 0.65	2.295	**0.03**
Tower of London (z-score, mean ± SD)	-0.52 ± 1.07	-1.18 ± 1.15	1.360	0.19
Composite Score (z-score, mean ± SD)	-1.37 ± 1.09	-2.43 ± 0.48	2.590	**0.02**
**CDSS**				
Depression Hopelessness factor (mean ± SD)	4.36 ± 2.47	4.38 ± 3.70	-0.014	0.99
Guilt-Self depreciation factor (mean ± SD)	1.29 ± 1.27	2.00 ± 2.39	-0.924	0.37
Early wakening factor (mean ± SD)	0.64 ± 0.84	0.88 ± 1.36	-0.498	0.62
Total score (mean ± SD)	6.36 ± 3.87	7.13 ± 5.16	-0.390	0.69

BACS: Brief Assessment Cognition Schizophrenia; CDSS: Calgary Depression Scale for Schizophrenia; CGI: Clinical Global Impression rating scales; LAI: Long Acting Injectable; n: number; PANSS: Positive and Negative Syndrome Scale, SD: standard deviations; tDCS: transcranial Direct Current Stimulation.
